# Percutaneous Kidney Biopsy: Indications, Technique, and Peri-Procedural Management—A Narrative Review

**DOI:** 10.3390/diagnostics16142290

**Published:** 2026-07-22

**Authors:** Saud Abdulelah O. Alsaleh, Mariam Omar Shikshok, Ahmed Abdel-Aal, Ammar Almehmi

**Affiliations:** 1 Department of Medicine and Radiology, University of Alabama at Birmingham, Birmingham, AL 35233, USA; aalmehmi@uabmc.edu; 2 Faculty of Medicine, University of Aleppo, Aleppo 12212, Syria; mariamshikshok@gmail.com; 3 Department of Interventional Radiology, The University of Texas Health Science Center at Houston, Houston, TX 77030, USA; ahmed.k.abdelaal@uth.tmc.edu

**Keywords:** kidney biopsy, percutaneous renal biopsy, interventional nephrology, ultrasound guidance, complications, hematuria

## Abstract

Percutaneous kidney biopsy (PKB) is an essential tool in the nephrologist’s armamentarium that plays a major role in establishing the diagnosis, monitoring response to interventions and providing prognostication. Over the last few decades, the technique of PKB has evolved from a blind procedure with a high complication rate to an image-guided procedure with substantially reduced but still clinically relevant complication rates, with major bleeding reported in approximately 1–6% of biopsies in contemporary series. With appropriate pre-procedure planning, patient selection, and experienced operators, PKB generally has a low rate of major complications and a high diagnostic yield, while clinicians must remain vigilant for bleeding and other adverse events. In this narrative review, we summarize current indications, contraindications, technical considerations, and complication mitigation strategies for PKB, integrating contemporary guideline recommendations and key observational studies to support bedside decision-making. Further, we review details pertaining to peri-procedure protocols. Special populations that require PKB such as morbid obesity, pregnancy, elderly, single kidney and anatomic abnormalities are discussed as well. Alternative approaches to performing PKB, when PKB is infeasible or challenging, are briefly covered in this review.

## 1. Introduction

The global burden of kidney disease represents a major public health challenge [1]. Percutaneous kidney biopsy (PKB) is an essential tool for diagnosis, prognostication, and monitoring of renal disease [2,3,4,5]. Over the last century, PKB has evolved from a blind technique to a precise, image-guided procedure using automated spring-loaded devices, which has significantly improved its safety profile and diagnostic yield [2,6,7,8,9,10,11,12]. The goal of this paper is to present a comprehensive review of PKB role in clinical practice.

Methods and scope of this review. This article is designed as a narrative, clinically focused review rather than a formal systematic review. We searched PubMed/MEDLINE and Embase for English-language articles published between January 2000 and May 2026 using combinations of terms such as “kidney biopsy,” “renal biopsy,” “complications,” “anticoagulation,” “obesity,” “pregnancy,” and “transjugular kidney biopsy,” and we also reviewed key guideline documents and society statements from major nephrology and interventional radiology organizations. We prioritized adult human studies, high-quality guidelines, meta-analyses, and large observational cohorts, with selective inclusion of earlier seminal studies where needed to provide historical context. Case reports and small series were included only when they addressed specific technical or high-risk scenarios (such as, solitary kidney or complex anatomy) not covered by larger datasets. Recommendations in this review emphasize consensus guidelines when available, supplemented by observational data and expert practice patterns where evidence is limited.

### 1.1. Indications of Kidney Biopsy

The decision to perform PKB balances diagnostic benefit with procedural risk. Primary indications include unexplained acute kidney injury (AKI), nephrotic syndrome, glomerular hematuria, and systemic diseases such as lupus nephritis or vasculitis, where histological classification dictates management [5,6,13,14]. In kidney transplantation, allograft biopsy remains the gold standard for diagnosing rejection and graft dysfunction [15,16]. Additional details are summarized in Table 1.

### 1.2. Contraindications to Kidney Biopsy

Kidney biopsies are often elective procedures and, as such, should be carried out in the safest possible setting. Contraindications to PKB can be classified into absolute and relative categories (Table 2).

#### 1.2.1. Absolute Contraindications

1. *Uncontrolled bleeding diathesis*: Although institutional cutoffs vary, evidence and guidelines suggest safety thresholds to reduce post-PKB hemorrhage. Thrombocytopenia independently increases bleeding risk; platelet counts <120,000/µL are associated with a >6-fold higher risk of major complications. The SIR recommends platelet transfusion for kidney procedures when platelets are <50,000/µL [2,5,22,28], whereas many nephrology practices target > 100,000–120,000/µL for elective PKB [2,5,22,28]. INR > 1.5 or aPTT > 40–45 s is generally considered a contraindication [1,4,5,18,28,29,30].
2. *Uncontrolled severe hypertension*: Elevated blood pressure markedly increases post-biopsy bleeding risk. SBP > 160 mmHg nearly doubles major bleeding (10.7% vs. 5.3%) [8,18], while SBP > 170 mmHg is associated with a 23-fold higher risk, suggesting a non-linear relationship [8]. DBP > 90–100 mmHg increases complication risk 7.6-fold [8]. This may reflect impaired vasoconstriction of rigid, hyalinized vessels under high hydraulic pressure [9,29].
3. *Uncooperative patient*: Patient immobility and breath-holding are essential to prevent renal injury during biopsy. If cooperation is not possible, PKB should be performed under general anesthesia for safety.

#### 1.2.2. Relative Contraindications

**Figure 1:**
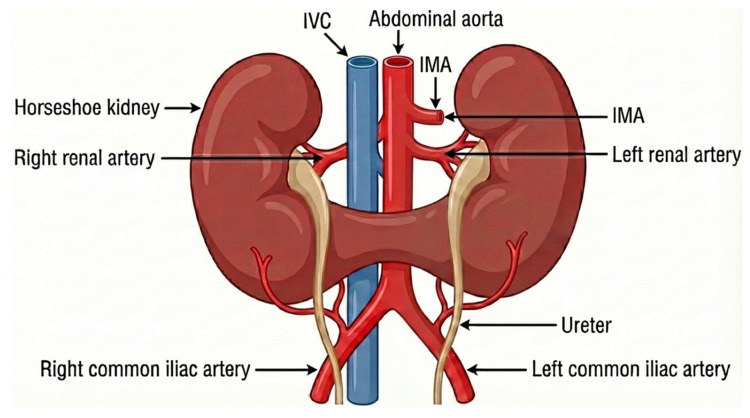
Horseshoe kidney anatomy and typical arterial supply. Anterior schematic of a horseshoe kidney (HSK) demonstrating lower-pole fusion with a midline parenchymal isthmus, positioned low in the abdomen (approximately L3–L5) beneath the origin of the inferior mesenteric artery (IMA). The isthmus lies anterior to the abdominal aorta and inferior vena cava (IVC), and the renal collecting systems are often malrotated with anteriorly oriented hila/pelves. The arterial supply shown is simplified to the typical pattern: single right and left renal arteries arising from the abdominal aorta, with a single midline aortic branch supplying the isthmus (not shown in figure).

1. *Solitary Native Kidney*: Solitary native kidney. Historically, biopsy of a solitary native kidney was considered an absolute contraindication due to concern that life-threatening hemorrhage could necessitate nephrectomy and result in an anephric state [18]. However, contemporary series demonstrate that solitary-kidney biopsy can be performed safely in experienced hands, with high technical success and low rates of major complications [23]. In modern practice, a solitary native kidney is therefore best regarded as a relative contraindication rather than an absolute one, with the decision individualized based on operator expertise, anticipated diagnostic yield, and consideration of alternative approaches (e.g., transjugular or laparoscopic biopsy).
2. *Small Kidney Size*: Kidneys < 9 cm are a relative contraindication due to perceived lower yield and higher complication risk [13]. However, recent data show complication rates in small kidneys (mean 7.56 ± 0.33 cm) are comparable to non-small kidneys with good diagnostic yield, and biopsy findings changed management in 64% of patients—suggesting size alone should not preclude biopsy when the diagnosis remains unclear [24,25].
3. *Uremia*: Uremia increases bleeding risk via platelet dysfunction (impaired aggregation and platelet–vessel wall interaction). BUN > 60 mg/dL is associated with a 9-fold higher bleeding risk [8]. The role of intranasal/subcutaneous desmopressin to mitigate this risk remains controversial [31].
4. *Anatomic Abnormalities*: (a) The presence of multiple bilateral kidney cysts (such as in polycystic kidney disease) interferes with obtaining a representative cortical sample and increases the bleeding risk [13,18]. Biopsy of a kidney mass is generally avoided to prevent tumor seeding or hemorrhage [32]. (b) Hydronephrosis should be relieved prior to biopsy to avoid infection and urine leakage. (c) Active pyelonephritis, perinephric abscess, or skin infection at the biopsy site are contraindications. (d) Horseshoe kidney poses hemorrhage risk due to an isthmus and variable anomalous vasculature (often near the aorta; Figure 1); CT guidance is recommended for anatomic delineation. If percutaneous access is unsafe, TJKB or laparoscopic biopsy are preferred alternatives [2,5,33].

### 1.3. Pre-Procedural Assessment and Patient Preparation

(i) History and physical exam: A thorough personal and family history of bleeding diatheses is required [34,35]. A comprehensive medication review is essential, especially the use of anticoagulants, antiplatelet agents, non-steroidal anti-inflammatory drugs (NSAIDs), and over-the-counter supplements (such as omega-3 fatty acids) that may impair platelet function [2,7,18]. The physical examination should assess the patient’s ability to cooperate during procedure and the integrity of the skin overlying the biopsy site [12].
(ii) Pre-kidney biopsy laboratory tests: In general, comprehensive metabolic panel, complete blood count, INR and aPTT are obtained [18,34]. A blood type and antibody screen (type and screen) are routinely recommended to facilitate rapid crossmatching if a blood transfusion is needed.
(iii) Blood Pressure: A blood pressure of <140/90 mmHg is recommended [18,35]. If needed, elevated blood pressure can be treated with oral or intravenous antihypertensives prior to biopsy.
(iv) Medication management: Strict management of antithrombotic agents is crucial. A summary of commonly used discontinuation and resumption intervals for antiplatelet and anticoagulant agents is provided in Table 3; these intervals synthesize available guideline recommendations and prevailing expert practice patterns rather than prescribing a single mandatory protocol. Timing should be individualized according to renal function and drug clearance, baseline bleeding risk (including anemia, uncontrolled hypertension, and uremia), thrombotic indication and urgency (for example, mechanical heart valves, recent venous thromboembolism, coronary stents), and whether the biopsy is elective or urgent. In general, agents are withheld long enough to achieve acceptable coagulation parameters and then resumed between 24 and 72 h post-biopsy in the absence of bleeding, with earlier or delayed reinitiation for very high thrombotic- or bleeding-risk scenarios.

### 1.4. Biopsy Technique and Equipment

The evolution of the PKB from a blind aspiration procedure to a precise, image-guided intervention has significantly enhanced its diagnostic yield and procedural safety. Details pertaining to the imaging modalities, patient positioning, and equipment selection are discussed below.

## 2. Imaging Modality and Guidance

Real-time ultrasound guidance is considered the gold standard to perform PKB and is associated with higher diagnostic yield and lower complication rate as compared to blind techniques [10,12,18,33]. Both high-resolution convex-array transducer and color Doppler are utilized pre-procedure to identify the major vessels and assess the projected needle path to avoid inadvertent injury to adjacent structures.

Computed Tomography (CT) guidance is generally reserved for cases in which the ultrasound visualization is inadequate, such as morbid obesity or complex anatomical variations (including horseshoe kidneys or multiple cysts) [2,38].

## 3. Patient Positioning

**Figure 2:**
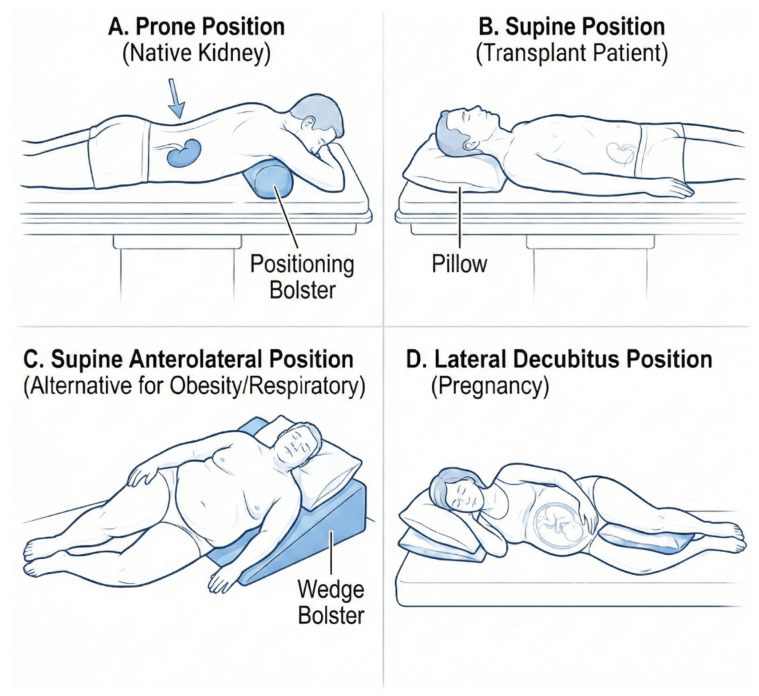
Patient positioning options for percutaneous kidney biopsy. Four common positioning strategies are illustrated to optimize access and procedural safety based on clinical context. (**A**) Native kidney biopsy performed in the prone position with a pillow or sandbag placed under the lower chest and upper abdomen to gently reduce lumbar lordosis, improve posterior lower-pole access, and stabilize the kidney against the posterior abdominal wall. (**B**) Kidney transplant biopsy performed in the supine position, allowing direct access to the allograft located in the iliac fossa. (**C**) Supine anterolateral position (SALP), in which partial lateral rotation in the supine position creates an acoustic and needle access window without the need for full prone positioning, particularly useful in patients with obesity or respiratory compromise. (**D**) Pregnancy-adapted positioning using lateral decubitus or seated posture to avoid aortocaval compression while maintaining safe biopsy access.

Proper positioning that accommodates the patient’s anatomy and body habitus is an important step in kidney biopsies (Figure 2).

- For native kidney biopsy, the prone position is considered the standard approach, with a firm pillow or sandbag placed beneath the lower chest and upper abdomen. This support gently reduces lumbar lordosis, elevates the posterior lower pole of the kidney toward the dorsal body wall, and improves stability of the kidney against the posterior abdominal wall while avoiding excessive intra-abdominal compression [10,18].
- Kidney transplant patients are placed in the supine position [10,18] owing to the superficial location of the allograft in the iliac fossa.
- In patients with morbid obesity or respiratory compromise, the supine anterolateral position is a safe and effective alternative that improves kidney access and visualization and reduces respiratory movement [7,18].
- In pregnant patients, to avoid compression of the gravid uterus, patients may be biopsied in the lateral decubitus or sitting upright position [7,18].
- In practice, the degree and location of support are individualized; in patients with marked obesity or limited respiratory reserve, the pillow height may be reduced or shifted slightly cranially to avoid excessive abdominal compression, whereas in pregnancy lateral decubitus or seated positioning is used to avoid aortocaval compression while maintaining a stable acoustic window (Figure 2).

## 4. Needle Selection: Type and Gauge

**Figure 3:**
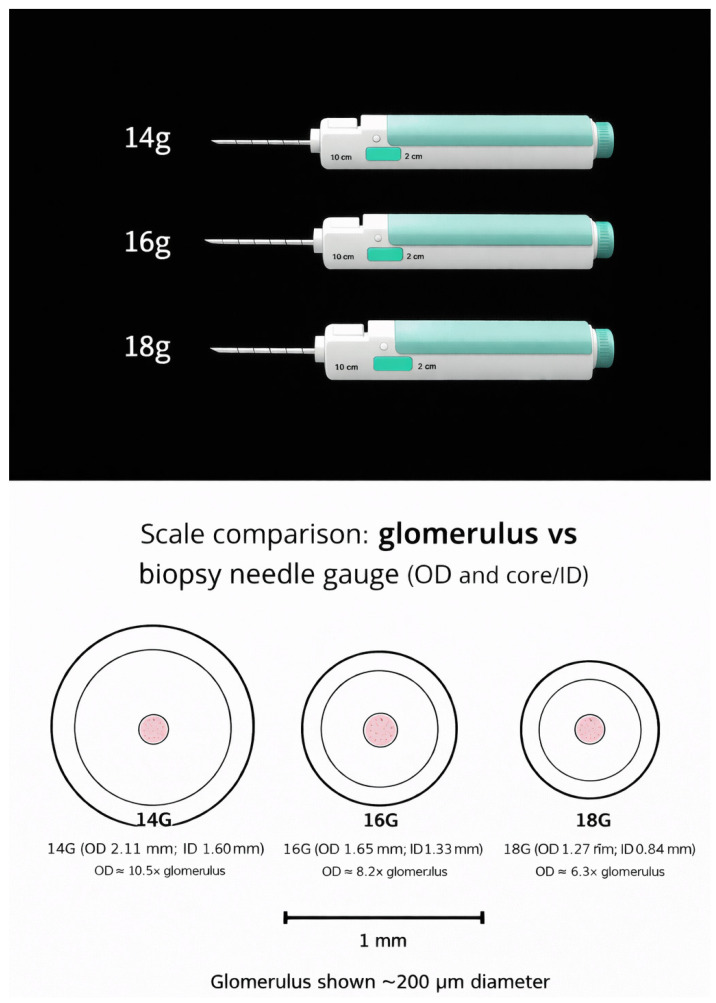
Scale comparison of automated renal biopsy needle gauges and the glomerulus. The **upper panel** illustrates commonly used automated spring-loaded renal biopsy devices in 14-gauge (14G), 16-gauge (16G), and 18-gauge (18G) configurations. The **lower panel** provides a to-scale cross-sectional comparison of each needle’s outer diameter (OD) and inner/core diameter (ID) relative to a representative human glomerulus (≈200 µm in diameter). Concentric rings depict the needle OD (outer circle) and tissue-capturing core/ID (inner circle), with the glomerulus shown centrally for reference.

Automated spring-loaded core biopsy devices are widely used in most medical centers due to procedural efficiency and reproducible tissue cores [10,26]. Different needle gauges are used in clinical practice as detailed below (Figure 3).

While 14-gauge needles provide more glomeruli, they are associated with higher transfusion rates (2.1% vs. 0.4%) compared to 16-gauge needles [11]. Therefore, 16-gauge needles are widely preferred as they offer the optimal balance between diagnostic yield and safety [2,8,11,26,35,38,39,40]. Additionally, 18-gauge needles are often reserved for high-risk cases but may result in inadequate sampling [17,40,41,42,43].

## 5. Trajectory and Approach

**Figure 4:**
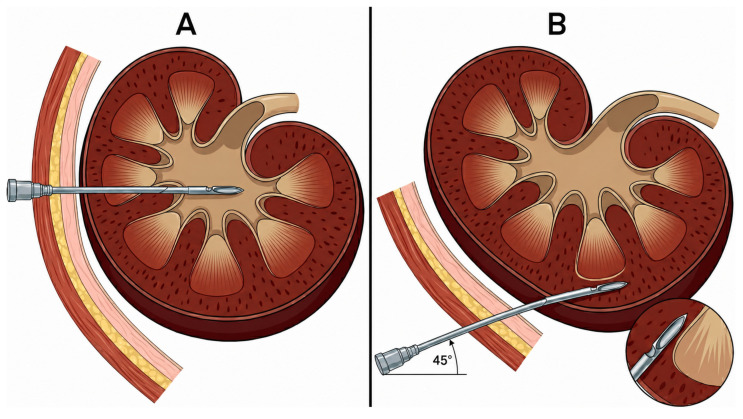
Biopsy needle trajectory. Comparison of needle paths during percutaneous kidney biopsy. (**Panel A**): An incorrect, steep transmedullary trajectory that traverses the medulla toward the renal sinus/hilum, increasing the risk of vascular and collecting system injury. (**Panel B**): The correct tangential cortical trajectory, typically ~45–60° relative to the renal surface, in which the needle remains entirely within the renal cortex and directed away from the hilum and medulla. This approach maximizes glomerular sampling while minimizing bleeding and other procedure-related complications.

The most important technical determinant of both diagnostic yield and safety is maintaining a needle path that stays entirely within the renal cortex, maximizing glomerular sampling while minimizing any injury to the intrarenal vessels, medullary pyramids, renal sinus, or the hilum [38,44]. Accordingly, the cortical tangential approach is favored as the needle is directed through the cortex (often described as ~45–60° relative to the renal surface) away from the hilum and sparing the vascular-rich medulla (Figure 4). This technique reduces the risk of significant hemorrhage and arteriovenous fistula formation [38,44].

For native kidneys, the lower pole of the left kidney is most common target for biopsy due to its accessibility and distance from major organs and vessels [10,18]. For transplant kidneys, the upper pole is often selected to avoid the renal pelvis and ureter, though the anterior or anterolateral aspects are also accessible.

Practically, the target should be a region of relatively thicker parenchyma, avoiding any cortical scars or markedly thinned cortex to increase the tissue yield and mitigate the bleeding risk. When approaching very tangentially, the introducer/needle may slide off the capsule; a useful maneuver described in the literature is to briefly steepen the angle as the tip touches the capsule to “bite” into it, then return to the planned tangential angle [38].

It is worth noting that transplant biopsies have unique technical considerations; the very early post-transplant biopsies carry a higher bleeding risk due to graft friability; older grafts can develop pericapsular fibrosis that may require advancing the needle tip slightly below the capsule before firing to avoid “skiving” off the capsular surface [16,18].

## 6. Needle Guides vs. Freehand Technique

The biopsy can be performed using a freehand technique or with a needle guide attached to the ultrasound probe. The use of a needle guide ensures the needle follows a predetermined track, which can enhance visualization and precision, improving biopsy adequacy and reducing minor complication rates in less experienced operators [9,45]. It is our experience that the use of freehand technique that keeps the needle tip within the ultrasound plane allows for greater flexibility in adjusting the needle path in real-time and is associated with minimal bleeding risks and excellent histologic yield [10].

## 7. Coaxial Technique and Trocars

**Figure 5:**
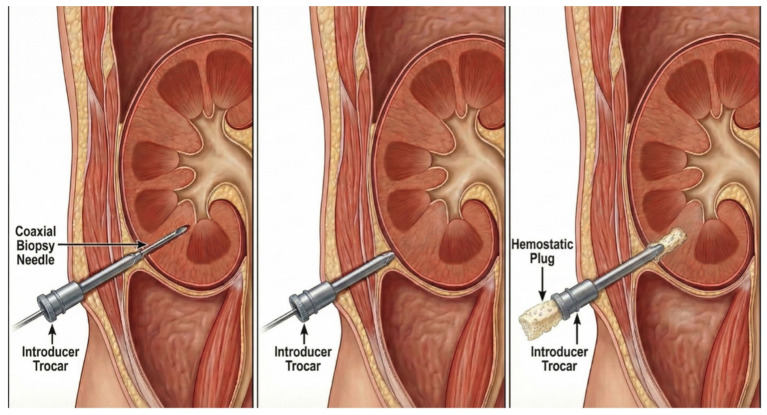
Coaxial technique for percutaneous kidney biopsy. Illustration of a coaxial biopsy system using a larger-gauge introducer trocar/sheath to access the renal cortex through a single capsular puncture. The cutting biopsy needle is advanced through the stationary introducer, allowing multiple cortical sampling passes and redirection without repeated skin or capsular entry. Following tissue acquisition, the introducer can be used to deliver a hemostatic agent into the biopsy tract to reduce post-procedural bleeding.

The coaxial technique utilizes a larger gauge introducer needle (trocar) through which the cutting biopsy needle is passed (Figure 5). This allows for multiple passes to be made through a single capsular puncture, potentially reducing trauma to the renal capsule and surrounding tissues [8,38]. This technique allows injection of a hemostatic agent (Figure 5) directly into the biopsy tract to reduce the risk of bleeding in high-risk patients [7,27,38].

## 8. Number of Passes

To mitigate complication risks, the number of biopsy passes should be restricted to the minimum required for diagnosis—typically 2 to 3 cores [8,12,43]. As such, exceeding 4 to 5 passes significantly increases the incidence of hemorrhage [8,19,45]. The utilization of on-site specimen evaluation, such as a dissecting microscope or pathologist review, is an effective strategy to limit the number of passes as this strategy confirms the specimen sufficiency in real-time.

## 9. Sample Adequacy

The required number of glomeruli is heavily dictated by the nature of the underlying pathology. Generally, a tissue sample that contains at least 10 glomeruli is considered the minimum requirement for a representative biopsy of a native kidney. In diffuse parenchymal diseases, the diagnosis can be established with as few as one or two glomeruli. However, in focal kidney diseases, a total of 15 to 20 glomeruli is believed to be optimal. Statistical modeling has demonstrated that a biopsy containing only 10 glomeruli carries a 35% probability of missing a focal lesion if the disease affects 10% of the glomeruli. Increasing the yield to 20 glomeruli reduces this probability of missing the lesion to approximately 12% [2,35].

Criteria for kidney allograft biopsies are more standardized. The Banff 97 classification defines an adequate specimen as one containing at least 10 glomeruli and at least two arteries to allow for the assessment of rejection and vasculopathy [40,43,46].

### 9.1. Post-Biopsy Monitoring

Post-procedural monitoring of PKB is critical for early detection of the complications. Most protocols focus on hemodynamic stability, surveillance of the puncture site, and monitoring of hemoglobin levels. In practice, a risk-stratified approach is useful, differentiating low-risk outpatient native biopsies, higher-risk native biopsies (for example, AKI or advanced CKD in hospitalized patients), transplant biopsies, and patients in whom anticoagulation is restarted early.

Major bleeding complications (such as hypotension and gross hematuria) usually manifest early after PKB. Although most studies indicate that 57–85% of these complications are identified within the first 4 to 8 h post-biopsy [1,17,35,41], approximately 9–11% of complications occur after 24 h of the biopsy [35,41]. Of note, delayed bleeding may occur even days to weeks after the biopsy, particularly after the resumption of anticoagulation or arteriovenous fistula rupture [12,42].

Traditionally, most patients undergoing PKB were observed in the hospital for 24 h after the procedure. This practice was driven by data indicating that about 33% of complications occurred after 8 h, up to 91% of complications were identified within 24 h [7,17,41]. However, more recent evidence suggests that an observation period of 4 to 8 h is sufficient for the majority of patients, particularly those with stable vital signs and no immediate complications [1,17,28,47,48]. For transplant kidney biopsies, which generally carry a lower risk of bleeding, observation periods as short as 4 h have been deemed safe [1,28]. In hospital settings, biopsies performed for AKI populations often carry a higher risk for complications, necessitating longer observation periods [19,49].

### 9.2. Post-Procedural Monitoring Protocol

Following the procedure, the patient is typically placed on strict bed rest in the supine position for 4 to 6 h to facilitate tamponade at the biopsy site [1,28,47,50]. Monitoring includes frequent vital sign measurements (e.g., every 15 min for the first 2 h, and then hourly); collecting the voided urine specimen; and measuring both hemoglobin and hematocrit levels at 4 to 6 h post-biopsy. A drop in Hgb of <1 g/dL is commonly seen in about 50% of uncomplicated biopsies, but a drop >2 g/dL may signal a major complication [7,30,35].

### 9.3. Risk Stratification

Risk-stratified monitoring. For low-risk native kidney biopsies performed electively in outpatients with controlled blood pressure (≤140/90 mmHg), adequate hemoglobin, normal coagulation parameters, and no major comorbidities, a 4–8 h observation period with frequent vital signs, assessment of flank pain, gross hematuria, and a single hemoglobin/hematocrit check is generally sufficient when early recovery is uneventful. In contrast, higher-risk native biopsies including patients with AKI, advanced CKD, uncontrolled hypertension, baseline anemia (for example, hemoglobin < 10 g/dL), or coagulopathy warrant prolonged observation (often 12–24 h), repeat hemoglobin measurements, and a lower threshold for post-biopsy imaging and inpatient monitoring. Transplant kidney biopsies typically have a lower bleeding risk, and several centers safely use observation periods as short as 4 h in otherwise low-risk outpatients, with more intensive monitoring reserved for early post-transplant biopsies or patients restarted on anticoagulation. Patients in whom anticoagulation or dual antiplatelet therapy must be resumed early (for example, recent coronary stent or venous thromboembolism) should be managed as high risk, with extended observation, delayed full-dose reinitiation when feasible, and careful coordination with the prescribing specialty [11,19,20,26].

### 9.4. Routine Post-Biopsy Ultrasound (US)

The routine use of US with Doppler after PKB remains a subject of debate [28]. While perinephric hematomas are detected in 60% to 90% of cases on routine imaging performed immediately after biopsy, these are often small and clinically silent [26,30,51]. Whereas the presence of a hematoma at 1 h post-biopsy has a low positive predictive value (43%), the absence of a hematoma at 1 h carries a high negative predictive value (95–100%) for late major hemorrhagic complications [51]. Hence, post-procedure US is not performed routinely for asymptomatic patients. On the other hand, those with severe flank pain, hemodynamic instability, or a significant drop in hemoglobin require immediate imaging.

### 9.5. Complications

The use of real-time ultrasound guidance and automated spring-loaded devices has substantially improved the safety profile of PKB. The rate of complications varies significantly based on the definition used (e.g., whether asymptomatic hematomas detected on surveillance imaging are included) and the patient population (native vs. transplant, inpatient vs. outpatient). The complication rates in a large meta-analysis of 118,000 biopsies were flank pain (4.3%), perinephric hematomas (11%), macroscopic hematuria (3.5%), bleeding requiring blood transfusions (1.6%), and death attributed to native kidney biopsy (0.03–0.06%) [52].

Generally, minor complications (those that resolve spontaneously without intervention) are seen in 6–15% of cases, while major complications that require intervention are seen in approximately 1–6.6% of biopsies [35,41].

*Bleeding*: this is the most common complication of PKB that typically manifests as hematuria or perinephric hematoma. Transient gross hematuria occurs in approximately 1.9–3.5% of cases and usually resolves spontaneously [11,53]. Clot retention with subsequent urinary tract obstruction is rare (0.3%) and requires immediate intervention to relieve the obstruction [11].

Perinephric hematomas are frequently detected on routine post-biopsy imaging (60–90%) but are rarely clinically significant. [30,41,51]. Symptomatic hematomas causing flank pain occur in 2.5–4.3% of patients [26,52]. while major hematomas requiring intervention are rare (0.9–5%) [6,28,46,54,55]. Page kidney, a rare complication involving compression-induced ischemia, requires immediate decompression. Notably, Page kidney has been reported more frequently following kidney allograft biopsy, it is rarely encountered after native kidney biopsy [7,8].

*Blood transfusion*: this is the most frequent major intervention, occurring in 0.9–1.6% of outpatient biopsies, 3.3% of transplant biopsies and 4.3–5.7% among hospitalized patients [11,19,52]. Importantly, the rate of transfusion is more common when larger needles are used for kidney biopsy [11]. In rare occasions, transcatheter arterial embolization is required (0.3–0.6%) to control the bleeding after kidney biopsy [11,52]. The need for nephrectomy to control life-threatening hemorrhage is extremely rare (0.06%) in modern practice [8,11].

*Arteriovenous fistula (AVF) formation*: The majority of AVFs that can occur after kidney biopsy [7,56,57] are asymptomatic and resolve spontaneously [57,58]. In rare occasions, arterial embolization is required to manage symptomatic large AVFs that are associated with persistent hematuria, hypertension, or high-output heart failure.

*Non-hemorrhagic complications*: Infectious complications, such as perinephric abscess and sepsis, are rare with adherence to strict sterile technique [10]. Injury to the collecting system or adjacent organs is rarely encountered nowadays with the use of imaging-guided biopsy.

### 9.6. Kidney Biopsy in Special Populations

#### 9.6.1. Transplant Kidney Biopsy

Transplant kidney is typically located superficially in the iliac fossa, making it easily accessible for effective manual compression after the biopsy. As such, the overall complication rate in transplant biopsies is lower than the native ones (3.9% vs. 6.5%), and is associated with lower transfusion rate compared to native kidneys (3.3% vs. 5.2%) [56]. However, the incidence of AVF is higher in allografts, detected in up to 10–16% of cases when routine Doppler monitoring is used, though the vast majority are asymptomatic and resolve spontaneously [57,58]. Biopsies performed within 1 week of transplantation carry substantially increased risk (311%) and should be deferred if clinically possible [16].

#### 9.6.2. Elderly Patients (≥60 Years)

With increasing prevalence of kidney disease in the aging population, biopsies in the elderly (typically defined as >60 years) are becoming more common. Despite concerns regarding multiple comorbidities, the evidence suggests that advanced age does not increase the risk of major complications [2,8]. Some evidence suggests that kidney biopsy in the elderly population has a high diagnostic yield that could potentially alter the management course, especially in treatable conditions such as pauci-immune glomerulonephritis, amyloidosis, and membranous nephropathy [3,13,14].

#### 9.6.3. Obesity

Obesity poses a technical challenge in performing the PKB owing to the increased depth of the kidney and the subsequent poor visualization by ultrasound [8]. At times, the supine anterolateral position is recommended for obese patients to improve access and visualization and reduce respiratory movement (Figure 2) [7,18]. However, when a US-guided approach is unfeasible, CT guidance or a transjugular approach can be used. Interestingly, the rate of major complication is not substantially higher in obese as compared to non-obese patients when adequate imaging is obtained [8]. In fact, a study of over 1000 biopsies found that patients with a lower BMI (mean 25.5) were at significantly higher risk for major complications compared to those with a higher BMI (mean 27.3) [36].

#### 9.6.4. Pregnancy

Kidney biopsy in pregnancy is generally reserved for very specific clinical scenarios in which the diagnosis cannot be established by non-invasive testing and histologic diagnosis will affect the overall management prior to delivery (e.g., distinguishing lupus nephritis flare from preeclampsia). A systematic review of 197 biopsies during pregnancy reported a 7% overall complication rate (2% major, 5% minor), as compared to 1% observed in the postpartum [59]. Furthermore, the risk is not uniform throughout gestation as most of the major complications were observed almost exclusively between 23 and 28 weeks of gestation. Biopsies that are performed earlier (up to 21 weeks) or later (28 weeks to term) in the pregnancy had more favorable safety profiles [2,18,59]. Alternative positioning (lateral decubitus, sitting upright) may be used to avoid compression of the gravid uterus (Figure 2) [7,18]. Postpartum biopsy is preferred whenever clinically feasible, particularly for non-urgent indications.

### 9.7. Alternative Biopsy Approaches

While performing PKB under ultrasound guidance remains the standard of care, specific clinical scenarios (such as uncorrectable bleeding diathesis, morbid obesity, solitary kidney, or failed percutaneous attempts) necessitate alternative approaches. Alternative methods, including transjugular, laparoscopic, and rarely open surgical biopsies, can be utilized in these high-risk populations.

#### Transjugular Kidney Biopsy (TJKB)

The transjugular approach, first described in 1990, utilizes the internal jugular vein to access the kidney vein and obtain tissue from the kidney cortex through the venous system [37,60]. This technique is particularly indicated for patients with coagulopathies, thrombocytopenia, morbid obesity, or those requiring simultaneous liver and kidney biopsies [22,37]. The primary safety advantage of TJKB is that any accidental bleeding from the corticomedullary junction leaks back into the venous system rather than forming a perinephric hematoma. Furthermore, if capsular perforation occurs, immediate hemostasis can be achieved via embolization [37,60]. In a series of 256 TJKBs performed on patients, many of whom were on anticoagulation, the procedure showed a diagnostic yield and safety profile similar to percutaneous counterparts [61]. Diagnostic adequacy for TJKB that utilize 18- or 19-gauge side-cutting needles is reported between 74% and 98%, with good tissue quality [37,60,61]. Major complications occurred in approximately 1–5% of cases, with interventions required to control the bleeding in 1–2% of patients [37,61].

### 9.8. Laparoscopic and Open Kidney Biopsy

Laparoscopic kidney biopsy (LKB) serves as a viable alternative that is characterized by direct visualization of the kidney, allowing for precise sampling and immediate hemostasis through electrocautery or suturing. In a large series using the retroperitoneal approach, adequate tissue was obtained in 96% of procedures [62]. Open Surgical biopsy is rarely performed nowadays and largely reserved for cases in which other methods are contraindicated or unsuccessful [63]. In a retrospective study of 115 patients undergoing open biopsy, the mortality rate was 0.8%, with a 27% rate of minor complications [64]. Both open and laparoscopic methods are rarely indicated nowadays, require general anesthesia, and are associated with higher cost and longer recovery time compared to the standard percutaneous methods.

### 9.9. Future Directions

While real-time ultrasound remains the gold standard for performing PKB, emerging modalities include contrast-enhanced ultrasound that offers improved detection of post-biopsy bleeding, and fusion imaging (combining real-time ultrasonography with CT or MRI), which offers a better localization in patients with complex anatomy [2,18]. Pre-procedural planning is also being refined through simulation training, which has been shown to modify puncture sites or contraindicate biopsies in over 11% of cases, thereby enhancing safety [65].

## 10. Conclusions

Percutaneous kidney biopsy (PKB), performed under real-time ultrasound guidance using automated biopsy devices, is a safe and essential diagnostic tool in medical practice. Kidney biopsy when performed by experienced operators with appropriate patient selection and risk stratification, has a low major complication rate and provides valuable diagnostic and prognostic information guide clinical management in most patients. Thorough pre-procedure evaluation and risk stratification, real time image-guidance, on-site specimen adequacy assessment, and post-procedure monitoring are prerequisites for a successful kidney biopsy. Preventive strategies (including blood pressure control, peri-procedural adjustment of anticoagulants and antiplatelets, accurate needle trajectory, careful needle gauge selection, and limiting the number of passes) minimize complication rates. Careful approach with special populations (pregnancy, elderly, obesity, AKI, high-risk bleeding patients) allows for individualized decision-making to minimize complications. Operator experience remains a critical determinant of procedural success and safety.

## Figures and Tables

**Table 1:** Comprehensive Indications for Kidney Biopsy.

General Category	Specific Indication	Clinical Context
**Proteinuria &** **Hematuria**	**New-onset Nephrotic Syndrome (Adults)**	Indicated to determine the specific underlying glomerular disease (e.g., FSGS, membranous nephropathy, minimal change disease) to guide immunosuppressive therapy. Exception: Routine biopsy is increasingly avoided in cases with circulating anti-PLA2R antibodies, which strongly indicate primary membranous nephropathy [3,13,14,17].
	**Sub-nephrotic Proteinuria**	Indicated if proteinuria is sustained (>1–2 g/day), particularly if accompanied by hypertension, hematuria, or declining renal function [3,6,13,14].
	**Glomerular Hematuria**	Indicated when microscopic hematuria (with dysmorphic red blood cells/casts) coexists with proteinuria or an elevated serum creatinine. Note: Isolated microscopic hematuria with normal kidney function is generally not an immediate indication, as it often reflects processes like thin basement membrane disease or stable IgA nephropathy that could be observed clinically [6,13,18].
**Acute & Chronic** **Kidney Dysfunction**	**Acute Kidney Injury (AKI) of Unknown Origin or Non-recovering AKI**	Indicated for intrinsic AKI when pre-renal (hypovolemia) and post-renal (obstruction) causes have been ruled out, or when standard workups are unrevealing as well as when suspected (ATN) fails to resolve within the expected timeframe, helping to assess chronicity or identify a missed diagnosis [13,14,18,19,20].
	**Acute Nephritic Syndrome/RPGN**	Indicated urgently for rapidly progressive glomerulonephritis (RPGN) with active urinary sediment (e.g., cellular casts) to confirm diagnoses like ANCA-associated vasculitis or anti-GBM disease and assess the percentage of viable versus sclerotic glomeruli [5,13,21].
	**Unexplained Chronic Kidney Disease (CKD)**	Considered for establishing an etiology or determining prognosis (assessing irreversible fibrosis vs. active inflammation) before the patient reaches end-stage kidney disease. Usefulness is limited in advanced CKD with small, hyperechoic kidneys [3,5,6,13].
**Systemic & Metabolic Diseases**	**Systemic Lupus Erythematosus (SLE)**	Recommended for any sign of renal involvement to classify the stage of lupus nephritis and dictate immunosuppressive regimens [5,13,18].
	**Diabetic Kidney Disease with Atypical Features**	Routine biopsy is not required for typical diabetic nephropathy. Biopsy is indicated for atypical features: rapid deterioration of renal function, sudden onset of nephrotic syndrome, active urinary sediment, or absence of diabetic retinopathy, which suggests a non-diabetic renal disease [5,6,13].
	**Paraproteinemias & Amyloidosis**	To identify renal manifestations of systemic diseases such as multiple myeloma, monoclonal gammopathy, or amyloidosis [5,13,14,18].
	**Drug-Induced Nephrotoxicity**	Increasingly indicated for patients undergoing novel oncologic therapies (e.g., immune checkpoint inhibitors, VEGF inhibitors, tyrosine kinase inhibitors) who develop acute renal impairment [18].
**Renal Allograft (Transplant)**	**For-Cause Biopsies**	Indicated for primary non-function (to distinguish ATN from early rejection), rapid or progressive decline in graft function, or new-onset proteinuria/hematuria [5,8,15,16].
	**Protocol (Surveillance) Biopsies**	Routinely performed by some transplant centers at predetermined postoperative intervals (e.g., 3, 6, or 12 months) to detect subclinical rejection or early drug toxicity [5,8,15,16].

**Table 2:** Contraindications to percutaneous kidney biopsy and mitigation strategies.

Category	Contraindication	Practical Thresholds	Why It Matters	Mitigation/Alternatives
Absolute	Uncontrolled bleeding diathesis/uncorrectable coagulopathy	Common practice targets platelet count > 100,000–120,000/μL for elective biopsy; prolonged aPTT (>40–45 s or >1.2× control) is associated with increased bleeding risk [6,22].	Markedly increases risk of major hemorrhage, transfusion, and need for intervention	Correct reversible abnormalities; defer elective biopsy; consider transjugular/laparoscopic approaches when correction is not feasible
Absolute	Uncontrolled severe hypertension	Higher major bleeding risk reported with DBP ≥ 90 mmHg and when SBP > 170 mmHg [6,13,18].	High intrarenal perfusion pressure may impair hemostasis and increases bleeding risk	Optimize BP prior to biopsy; reassess on day of procedure; consider inpatient monitoring in higher-risk patients
Absolute	Uncooperative patient/inability to follow instructions	Inability to maintain position or cooperate with breath-holding/stillness during needle deployment [5,13].	Increases risk of needle misdirection and vascular/adjacent organ injury	Optimize analgesia/anxiolysis; consider monitored sedation or alternative approaches when cooperation cannot be ensured
Relative	Solitary native kidney	Single functioning kidney where complications could result in loss of renal function [2,6,13,18,23].	High-consequence complication risk even if absolute incidence is low	Careful risk-benefit assessment; consider alternative approaches and enhanced monitoring
Relative	Small hyperechoic kidneys/suspected advanced irreversible CKD	Historically: kidney length < 9 cm considered higher risk/lower yield [13,24,25].	Potentially lower diagnostic yield and less likelihood of changing management in end-stage scarring. More recent evidence suggests high yield and low complication rates.	Individualize; do not exclude solely on size if diagnosis remains unclear and would change management; ensure experienced operator and optimized imaging
Relative	Uremia/qualitative platelet dysfunction	Advanced uremia associated with increased bleeding risk [13,26,27].	Platelet dysfunction may increase bleeding risk despite normal platelet count	Optimize uremic milieu when feasible; consider center-specific hemostatic measures (e.g., desmopressin where used) and higher-intensity monitoring
Relative	Anatomic abnormalities and local infection	Multiple bilateral cysts, renal tumor/mass, hydronephrosis; active renal/perirenal infection or overlying skin infection [5,6,13].	Reduced ability to safely access cortex; increased risk of collecting-system injury or sepsis	Treat infection; relieve obstruction; consider CT-guidance or alternative approaches (transjugular, laparoscopic/open)
Relative	Horseshoe kidney (complex anatomy)	Aberrant vasculature and altered anatomic relationships [2,6].	Higher risk of vascular/adjacent structure injury	Pre-procedure imaging/planning; consider transjugular renal biopsy or laparoscopic biopsy

**Table 3:** Peri-biopsy medication management.

Medication Class	Examples	Typical Hold Before Biopsy	Notes/Special Situations	Typical Resume After Biopsy	References
Antiplatelet (COX inhibition)	Aspirin	5–7 days	Some data suggest continuing low-dose aspirin may not significantly increase major bleeding risk in high cardiovascular-risk patients. In dual antiplatelet therapy, a common compromise is to hold clopidogrel while continuing aspirin [2,5,8,22].	Typically 7–14 days to minimize delayed hemorrhage; some protocols allow 1–2 days in high thrombotic risk [2,5,8,22].	[2,5,8,22]
Antiplatelet (P2Y12 inhibition; thienopyridines)	Clopidogrel (and other thienopyridines)	5–10 days (often ≥7 days)	A washout period of ≥7 days is standard in many centers to allow platelet function recovery. Consider holding this agent preferentially if dual therapy [18,22].	Typically 7–14 days; may resume 1–2 days in high thrombotic risk per some protocols [18,22].	[18,22]
NSAIDs	Ibuprofen, naproxen, etc.	3–7 days	Held to allow normalization of platelet function.	Typically 7–14 days to minimize delayed hemorrhage [5,22,36].	[5,22,36]
Vitamin K antagonist	Warfarin	5–7 days (to achieve normal INR)	Heparin bridging may be required for high thrombotic risk patients.	Generally 12–24 h if no bleeding; some sources suggest waiting 48–72 h before full therapeutic dosing [2,5,13,22].	[2,5,13,22]
Direct oral anticoagulants (DOACs)	Apixaban, rivaroxaban, dabigatran, etc.	48–72 h, depending on kidney function	Timing should account for renal clearance.	Generally 12–24 h if no bleeding; some suggest waiting 48–72 h before full therapeutic dosing [5,18,37].	[5,18,37]
Parenteral anticoagulant (UFH)	Unfractionated heparin	4–6 h	Short half-life allows brief interruption.	6–72 h post-biopsy if no bleeding depending bleeding and thrombotic risk [2,5,13].	[2,5,13]
Parenteral anticoagulant (LMWH, therapeutic dose)	Enoxaparin, dalteparin	24 h	Hold therapeutic dosing 24 h pre-procedure.	24–72 h post-biopsy if no bleeding depending bleeding and thrombotic risk [2,5,18].	[2,5,18]
Herbal/OTC supplements affecting platelets	Omega-3 fatty acids; herbal products with antiplatelet effects	Discontinue prior to biopsy	May contribute to bleeding risk.	Resume after hemostasis is secure and bleeding risk is acceptable [7].	[7]

Abbreviations: COX, cyclooxygenase; DOAC, direct oral anticoagulant; LMWH, low-molecular-weight heparin; NSAID, nonsteroidal anti-inflammatory drug; UFH, unfractionated heparin; OTC, over the counter. Hold and resumption intervals vary across centers and should be individualized based on bleeding risk, thrombotic indication and urgency, and kidney function. Where available, the suggested ranges reflect published guideline statements; when evidence is limited, they represent commonly adopted expert practice. Clinicians should adapt these intervals in consultation with cardiology/hematology for patients at very high thrombotic risk.

## Data Availability

No new data were created or analyzed in this study. Data sharing is not applicable to this article.
